# Pennsylvania State Veterinary Medical Association

**Published:** 1891-11

**Authors:** W. H. Ridge

**Affiliations:** Secretary Pro Tem.


### SOCIETY PROCEEDINGS.

*Pennsylvania State Veterinary Medical Association.*—The Pennsylvania State Veterinary Medical Association held its Semi-annual Meeting at Wilkesbarre, September 8, 1891, in the County Medical Hall. Drs. Kooker, G. B. Rayner, Hart, Shaufler, T. B. Rayner, Hoskins, Goentner, Ridge, Foelker, Stanton, Timberman, Butterfield, Keil and Sallade were present. Visitors were Drs. C. H. Good, Kellar, Vanderbilt, Bloom and Walters.

Minutes of March meeting read and approved. Dr. Hoskins recommended having blank forms for applicants for membership, to facilitate finding the standing of applicant.

Applications for membership were made by Drs. Waugh, of Allegany City; Kellar, of Williamsport; Good, of Lockhaven, and Millar, of Prestentown; the Censors reported favorably and they were elected.

Report of Committee on Legislation, Dr. Hoskins Chairman: "As your Chairman on Legislation I can only report the greatest inaction on the part of your committee for the past six months that has occurred since its original appointment. The causes for the lack of seeming duty is well-known to you all by this time, and we as your committee offer no excuse for its existence, inasmuch as the unfortunate amendment to our act, extending the time of registration of non-graduates had become a law before your committee knew of its existence, and was conceived and blossomed into the mature flower before one-half of those who constituted our legislators dreamed of its possible conception, I would not underestimate the responsibility resting upon those who knew of its probable birth and witnessed its entrance into life, and who did at the same time realize the necessity of informing your committee of its existence. The additional registrations so far as learned have not been excessive owing to the unusual number who had done so before the first limit of the time had expired. In Philadelphia but four new graduates have registered since its passage. At present we are pressing fearlessly, through the able assistance of Dr. Jas. A. Waugh, of Pittsburg, two prosecutions on the ground of false statements as to the possession of diplomas registered in Alleghany County. One of these, unfortunately, is a member of this association, much to our regret; but no leniency will be shown on this ground as he, above all others, should have known better and well deserves to be meted out a just punishment. What further action remains to be taken between now and January 1st., will entirely depend upon circumstances arising from time to time. We believe it is wise to remain quiet until that time lest an unfavorable decision against us would encourage a large number to register, which would necessitate a very great expenditure of time and money to eliminate."

Dr. Waugh will be furnished money for prosecution by the Association.

Dr. Hoskins presented a bill of $26.96 as costs in the Lancaster suit, which was ordered paid.

Report of Committee on Intelligence and Education, Dr. Huidekoper, Chairman, not being present the report was made by Dr. Sallade.

Mr. President and Gentlemen :

While nothing of special moment has taken place in veterinary practice since our last annual gathering nevertheless we cannot help congratulating the society and the profession in general upon the steady advance which Veterinary Science is making throughout our own country and abroad, and while much work might be desired in certain quarters in the way of organization and consolidation, still we have every reason to congratulate ourselves that there never was a time when so many able practitioners were at work, or so many learned publications bearing upon the profession issued from the press in various languages as to-day. The great impetus given to every department of science by the investigating, analytical spirit of the age has fairly taken possession of our particular field, and it is our glory in this land, and in this age to be the witnesses as well as adepts in a most useful science which was regarded as venerable among the most remote peoples, of whom we possess authentic history. The high antiquity of our profession, the supreme importance which it occupies in the physical economy of our modern society, its eminent respectability with all, at once constitute us the guardians of the physical welfare of those dumb animals which a Beneficent Providence has been pleased to surround man in order to aid his comfort and enjoyment; and thus in lending our intelligent efforts to the conservation of them we are indirectly laboring in the highest behests of society itself.

It is for these reasons that it well becomes this society to enlist in its behalf the best intelligence to be obtained and insist upon a thorough scientific course in some recognized veterinary school preparatory to entering upon legitimate practice, and in this particular your committee feels itself constrained to reiterate with emphasis the various salutary recommendations of former committees on intelligence and education of this society, and those especially which regard the duration and quality of the course of study to be pursued, as well as the character of the student himself. For it cannot for a moment be doubted that the learning and character of our practitioners will have, I may say, all to do with the shaping of the destiny and the attainments of the ultimate results of veterinary science; and it has for this reason, as well as on account of the close relationship existing between the methods of treating the various diseases ot man and beast, that the veterinarian should, in justice to his profession, emulate the character and rival the scientific attainments of his brethren of the sister medical profession. Upon the character, as well, of our practitioners will in no small measure depend our success when we knock at the door of the halls of Legislation and demand that protection and pecuniary encouragement which a science so far-reaching in its consequences to society merits, and which are, as its worthy representatives, will have reason to expect will be granted us. The age of the so-called Horse Doctor is gone forever.

We are instead an organized body of veterinary physicians, extending the benefits of our science to, and the prolonging our efforts in, a broader and less restricted field than heretofore. The horse is only one of the objects of our professional solicitude. The various species of cattle and other domestic animals, especially those whose flesh is used for human consumption, likewise belong to the sphere of our scientific investigation. The splendid scientific triumphs of recent years in bacteriological and tubercular pathogeny are matters of the most profound interest to the veterinarian and should engage his most earnest attention, and in connection with this, your committee would suggest that within the jurisdiction of this society where so many Jersey cattle are imported and raised, the special study of tuberculosis is absolutely necessary in order that we may be able to intelligently answer the many calls made upon us, since it is quite as certain that the great majority of this breed of cattle is infected with the dread disease in either a mild or malignant form, as it has recently been proved to a certainty that the milk and butter obtained from them carry with them the germ of tuberculosis. And while recommending this special study on account of the peculiar circumstances in which we find ourselves placed, in a general sense your committee would also recommend, in case the society still believes in the old adage that an ounce of preventive is worth a pound of cure the thorough study of prophylactics, always interesting, even when not applied; and thus having as it were, both ends of the disease, whatever it may be, within our grasp, we will be able the more readily to respond to the expectations of our patrons, and the sooner merit the title which it should be the ambition of each and every one of us to covet. *The Title of Fine Veterinarian.*

It was moved that the report be recorded and a vote of thanks extended to committee carried.

Dr. Zuill, Chairman of Committee on Sanitary Science, was not present. Dr. Timberman said they had no report to offer.

A Veterinary Association having been started in Pittsburg by Drs. Waugh, Carter and McNeil, it was moved that the Secretary send Dr. Waugh our best wishes, and ask him to send delegates to our next meeting.

Dr. G. Myer, having been charged with false registration, the charge was referred to Board of Censors at the March meeting. Dr. Sallade moved to expunge from the minutes that part refusing honorary membership to the gentleman he proposed, Carried. Dr. Hart moved that the Code of Ethics be read at every meeting, which was lost.

A communication was received, stating that Dr. Weber has been very sick with typhoid fever, but was improving slowly. Dr. Kooker spoke of Dr. Weber's misfortune in sickness during the last few years, and Dr. Hoskins moved that we tender him our sincere sympathies, which was carried.

Dr. Hart said that the President should give us a lecture to improve us in our actions toward one another, to stop this wrangling about the Code of Ethics.

The Treasurer was directed to notify the Secretary of the names of the delinquents, so that they can be acted on at the March meeting.

A paper "Results of Laryngitis," was read by Dr. Butterfield. Dr. Hoskins read a paper on "The Veterinarian as a Sanitarian." The Association extended a vote of thanks to Dr. Hoskins, and also ordered 1,000 copies printed for distribution.

In discussion Dr. Hoskins said, if we had sanitary inspectors we have no adequate sanitary laws; if we discover a case of glanders in the northwest part of Pennsylvania, we have to send to Dr. Bridge in Philadelphia before action can be taken. We have no member on the Board of Health, nor are we even recognized by it. The public should be educated up to the point that they will demand laws governing public funerals in case of contagious diseases. He stated that eighty children died in one town of diptheria, yet nothing was done to prevent the spread of the same.

Dr. Sallade said he had read a paper before the Association on "Cremation," and that he is also a leader in the "Bovine Association." This association had brought dollars into his pocket and aroused the people to the dangers in the food supply. He had rid the community in which he resides of tuberculosis in cattle; when a man buys a cow, the Bovine Association sends him to examine the cow, and if tuberculous it is at once dispatched. He diagnosed tuberculosis by slight rise in temperature, tenderness along the spine, emaciation, continued œstrum, and sibilant rales in lungs.

Dr. Ridge read a paper on Abscess of the Gutteral Pouches. *Vide.*, fol. 620.

Drs. Rayner and Hoskins thought it was originally an injury. While Dr. Shaufler thought as Dr. Ridge that it was pharyngitis at first then extending to pouch. As Dr. Shaufler had a case very similar, which he felt very sure was originally pharyngitis. Dr. Ridge cited a case of gangrene of anterior lobe of lung without being recognized by two good practitioners. Dr. Hoskins thought we might have gangrene of a small portion without being readily recognized, but thought we always have our increase in temperature, and as it is very difficult to auscult that portion of the lung, we have to be on our guard. Dr. Sallade thought the injury was an old one. A vote of thanks was extended for report of the case.

Dr. Hart said that fifteen years ago no veterinarian would dare acknowledge a mistake. But to-day they will call a brother practitioner in at any time, and talk over a case as true men should do. We are here to exchange ideas, it is seldom we find every one of the same opinion. Dr. Hoskins reported his visit to the New Jersey Association, and expressed himself as being well pleased with his visit, he spoke of receiving a letter from a veterinarian censoring him for encouraging quacks (non-graduates) to join our association. On the paper that the letter was written was illuminated a barn-yard scene, and explaining the place of graduation, etc. It is needless to say the writer received a very caustic reply, he said, "We made no mistake when we take in gentlemen if they are non-graduates, as some of our best workers are non-graduates.

President Kooker advised the local veterinarians to form associations. We should have these all over the State. Dr. Kellar reported a case of a mare, that had a discharge from the vulva, she had aborted a short time before, on examining vagina could not find anything to cause the trouble, but on dilating he found a hard substance inside when he removed four calculi. When he found the fifth so sharp he had to wrap a chamois around it before removing it. In the following July he saw a similar case, the hymen was not ruptured, found os entirely closed, dilated with forceps and removed four calculi. A short time after saw another case, of a Western mare which had a discharge per vulva, and was ruptured, when on examination he found a calculus in uterus. Stones resembled those lying in road, at first he thought they had been placed in the uterus to prevent evertion, a mare aborted a short time before, but when he came to the case of imperforated hymen, then he could not imagine how it gained entrance. The stones were sent to Dr. Kooker to be examined.

Dr. Good reported epidemic opthalmia in horses, around Lock Haven.

Three bills amounting to $28.75 in favor of Dr. Gladfelter were ordered to be paid. Delegates appointed to attend the United States Veterinary Association, are Drs. Fœlker, Harger, and Webster.

After which the Association adjourned, when Dr. Walters escorted the members and wives to the Nothingham Coal Mines, after going down and seeing the mining of coal they returned to their homes, feeling highly pleased at the meeting and kindness shown them by the Wilkesbarre Veterinarians.

W. H. Ridge, *Secretary Pro Tem.*

